# Anticancer effects of CKD-602 (Camtobell^®^) via G2/M phase arrest in oral squamous cell carcinoma cell lines

**DOI:** 10.3892/ol.2014.2648

**Published:** 2014-10-30

**Authors:** YOUNG-KYUN KIM, NA-YOUN KOO, PIL-YOUNG YUN

**Affiliations:** Department of Oral and Maxillofacial Surgery, Section of Dentistry, Seoul National University Bundang Hospital, Seongnam-si, Gyeonggi-do 463-707, Republic of Korea

**Keywords:** CKD-602, G2/M arrest, oral squamous cell carcinoma, apoptosis

## Abstract

CKD-602 (7-[2-(N-isopropylamino) ethyl]-(20S)-camptothecin, belotecan) is a synthetic water-soluble camptothecin derivative and topoisomerase I inhibitor that has been shown to exert a clinical anticancer effect on various types of tumor. In the present study, the anticancer effects of CKD-602 on the following three human oral squamous cell carcinoma (OSCC) cell lines originating from Korean cancer patients: YD-8 (tongue), YD-9 (buccal mucosa) and YD-38 (lower gingiva) were analyzed. The apoptotic proportion of the cells and cell cycle position were analyzed using flow cytometry. The expression of cell cycle regulatory proteins was detected by western blot analysis. CKD-602 was demonstrated to exert a time- and dose-dependent antiproliferative effect in all cell lines *in vitro*, however, susceptibility to CKD-602 at 72 h following treatment varied among the three cell lines, with 50% inhibition of cell viability at concentrations of 2.4 μg/ml for YD-8, 0.18 μg/ml for YD-9 and 0.05 μg/ml for YD-38. To investigate the underlying mechanism of the CKD-602 antiproliferative effect, a cell cycle-analysis was conducted in the three OSCC cell lines and CKD-602 treatment was observed to induce G2/M phase arrest. Furthermore, western blot analysis revealed that the expression levels of phospho-cdc2 (Tyr 15), cyclin A2 and cyclin B1 were increased in a time-dependent manner, following the administration of CKD-602. In the fluorescence-activated cell sorting analysis, the number of apoptotic cells was also increased in a dose-dependent manner following CKD-602 treatment of the OSCC cell lines. The results suggest that CKD-602 may inhibit the proliferation of OSCC oral cancer cells derived from samples from Korean patients by apoptosis and by G2/M phase arrest.

## Introduction

Squamous cell carcinoma is the most common type of malignant neoplasm of the oral mucosa and accounts for >90% of all intraoral malignant tumors, in Korea (1). In Korea, ~20% head and neck cancer is estimated to occur in the oral region (1). Despite gradual improvements in surgery, radiotherapy and chemotherapy treatments, the prognosis of patients presenting with locally advanced oral squamous cell cancer (OSCC) remains poor, with only a 50% survival rate over five years (2). Due to these poor survival rates and the severe functional impairment caused by surgery and radiation, the development of novel therapeutic strategies in the management of patients with advanced OSCC is urgently required.

Head and neck SCC cell lines in cultures are widely used to understand therapeutic development. However, the establishment of SCC cell lines is considered difficult and low success rates have been reported. Cell lines have previously been derived from patients who had received radiotherapy, with or without chemotherapy (3); however, radiotherapy and chemotherapy eliminate sensitive cell populations and may alter the *in vivo* progression and cell heterogeneity of the tumor (4). Genetic abnormalities in human cancer are markedly geographically dependent, and the cultural and environmental background of the patient are closely associated with the carcinogenic process. For example, oral cancer has been clearly associated with the presence of human papillomavirus HPV16 in Western countries, but not in Korea (5). In the current study, YD cell lines, which are newly established oral cancer cell lines originating from untreated oral tumors in Korean patients, were used (5). The YD cell lines were derived from untreated primary tumors of the tongue (YD-8), buccal mucosa (YD-9) and lower gingiva (YD-38), and the cell lines exhibited genetically different p53 statuses. The YD-8 cell line had a point mutation at codon 273 of exon 8, which is involved in the DNA-binding site, revealing its significance in p53 transcriptional activation; the GGT (arginine) sequence was replaced with CAT (histidine). This R273H mutation accounts for ~20% p53 missense mutations (6). The YD-9 and YD-38 cells did not have the p53 mutation; however, the p53 protein was positively expressed in the YD-9 cells but not in the YD-38 cells. As over half of all human cancers lose p53 function through mutation (7), investigation of the potential impact of p53 mutations on disease pathology and therapeutic response is important. Tumors with an inactive mutant p53 are aggressive and are commonly resistant to ionizing radiation and chemotherapy (8).

DNA topoisomerase I (Top1), an essential nuclear enzyme that controls and modifies the topological state of DNA in numerous cellular metabolic processes (9,10), serves as a target for screening anticancer agents (10–12). CKD-602 (7-[2-(N-isopropylamino) ethyl]-(20S)-camptothecin; belotecan), a Top1 inhibitor, is a novel, synthetic, water-soluble camptothecin derivative (13). Preclinical trials of CKD-602 have demonstrated that CKD-602 exerts antitumor activity against various human tumor cell lines, and that the results are equal or superior to those of camptothecin (13). In a previous study, CKD-602 was observed to exert an *in vitro* anticancer effect on three OSCC cell lines, A253 (submandibular gland), HSC-3 (tongue) and KB (oral mucosa) (14). In the present study, the potential effects of CKD-602 on cell viability in OSCC cell lines originating from oral cancer in Korean patients with genetically different p53 statuses was evaluated, as well as the mechanisms underlying the induction of cell cycle arrest and apoptosis.

## Materials and methods

### Reagents

CKD-602 (Chong Kun Dang Pharmaceutical Corp., Seoul, Korea) was dissolved in distilled water at 1 μg/ml, and stored as a stock solution in aliquots at −20°C until use. Final concentrations between 0.01 and 10 μg/ml CKD-602 were obtained by appropriate dilutions of the stock solution with RPMI 1640 medium (Gibco-BRL, Grand Island, NY, USA).

### Cell lines and cell culture

Three OSCC cell lines, YD-8 (60501; tongue), YD-9 (60502; buccal mucosa) and YD-38 (60508; lower gingiva) were used (4). All cell lines were obtained from the Korean Cell Line Bank (Seoul, Korea).

Each cell line was maintained in RPMI-1640 medium (Gibco-BRL), supplemented with 10% heat-inactivated fetal bovine serum (FBS, Gibco-BRL), 100 μg/ml streptomycin (Gibco-BRL) and 100 IU/ml penicillin (Gibco-BRL), as a monolayer under standard conditions (37°C, and in a humidified atmosphere of 5% CO_2_). To transfer or passage the cell lines, each confluent monolayer was washed with phosphate-buffered saline (PBS; Welgene, Daegu, Korea) and detached with a 0.05% trypsin/0.02% EDTA solution (Gibco-BRL).

### MTS viability assay

Cells at a density of 2×10^4^ cells/well in 100 μl RPMI with 10% FBS were added to the wells of a 96-well plate. The cells were treated with different concentrations (0.01, 0.1, 0.5, 1, 5 and 10 μg/ml) of CKD-602 for 24, 48 and 72 h. Control samples of each cell line were treated with medium only. For the viability assay, 20 μl/well CellTiter 96^®^ AQueous One Solution Reagent (MTS; Promega Corporation, Madison, WI, USA) was added. After 1 h incubation at 37°C in a humidified atmosphere of 5% CO_2_, the absorbance at 490 nm was recorded using an ELISA plate reader (Bio-Tek Instruments, Inc., Winooski, VT, USA) The assay was performed in triplicate with three independent experiments for each condition. The data from the treatment groups were normalized to those of the control samples and are presented as the mean ±standard error of the mean. The half maximal (50%) inhibitory concentration (IC_50_) values were calculated from the dose-response curve.

### Annexin assay

Apoptosis was quantified using fluorescein isothiocyanate (FITC)-Annexin V Apoptosis Detection kit I (BD Biosciences, San Jose, CA, USA) according to the manufacturer’s instructions. Briefly, the cells were plated at a density of 1×10^6^ cells/well in a 100 mm culture dish, treated with 0.1 and 0.5 μg/ml CKD-602 for 48 h, and harvested by centrifugation at 500 × g for 3 min. The cell pellets were resuspended in Annexin V binding buffer containing 140 mM CaCl_2_, 10 mM HEPES/NaOH and 2.5 mM MgCl_2_. FITC-conjugated Annexin V (5 μl) and propidium iodide (PI; 5 μl) were added to the cells, and the mixtures were incubated for 15 min at room temperature in the dark. The analyses were performed using a Fluorescence-Activated Cell Sorting (FACScan) instrument (Becton-Dickinson, Franklin Lakes, CA, USA). Annexin V- and PI-positive cells were considered to be apoptotic.

### Cell cycle analysis

The cells were plated at a density of 1×10^6^ cells/well in 100 mm culture dishes. The cells were treated with 0.02 μg/ml CKD-602 for 48 h, harvested by centrifugation at 500 × g for 3 min, washed twice in ice-cold PBS, fixed in 70% ethanol and stored at −20°C for a minimum of 1 h; subsequently, cells were washed with ice-cold PBS and resuspended in 500 μl PI/RNase Staining Buffer (BD Biosciences). The cell cycle position was evaluated by FACScan using an excitation laser set at 480 nm and a detection wavelength of 575 nm. A minimum of 10,000 events/sample was analyzed.

### Western blot analysis

CKD-602-treated and non-treated cells were suspended in RIPA buffer (Rockland, Gilbertsville, PA, USA) containing 5 μM AEBSF, 1.5 μM aprotinin, 10 μM E-64, 0.01 μM leupeptin and phosphatase inhibitors [1 mM sodium orthovanadate (Na_2_VO_4_; S6508; 1 mM sodium molybdate (Na_2_MoO_4_; M1003) 4 mM sodium tartrate dihydrate (S4797); 2 mM imidazole (I0125) all purchased from Sigma-Aldrich, (St. Louis, MO, USA)], and were placed on ice for 20 min. Following centrifugation at 4°C at 10,000 × g for 20 min, the cell supernatant was collected. The protein concentration was determined using a bicinchoninic protein assay kit (Pierce, Rockford, IL, USA). The whole lysate (20 μg) was resolved on a 10% or 12.5% SDS-PAGE gel, transferred to a polyvinylidene difluoride membrane (Bio-Rad, Hercules, CA, USA) by electroblotting, and probed with polyclonal rabbit anti-human β-actin (4967), monoclonal mouse anti-human p53 (9826), monoclonal mouse anti-human phospho-H3 (Ser 10; 3377), polyclonal rabbit anti-human cyclin B1 (4138), monoclonal rabbit anti-human cyclin A2 (4656), polyclonal rabbit anti-human cdc2 (9112), polyclonal rabbit anti-human phopho-cdc2 (Tyr 15; 9111), polyclonal rabbit anti-human Myt1 (4282) or polyclonal rabbit anti-human phospho H2AX (Ser 130; 9718) antibodies (1:1,000; Cell Signaling Technology, Inc. (Danvers, MA, USA) overnight at 4°C. The membrane was washed three times with 1× PBS and 0.05% Tween-20 for 15 min, and incubated for 1 h with a horseradish peroxidase-conjugated polyclonal horse anti-rabbit (7074) or polyclonal goat anti-mouse (7076) antibody. (1:2,000; Cell Signaling Technology, Inc.). The blot was developed using an enhanced chemiluminescence kit (Intron Biotechnology Inc., Seongnam, Korea).

## Results

### Effects of CKD-602 on cell viability of OSCC cell lines

**Figure 1:**
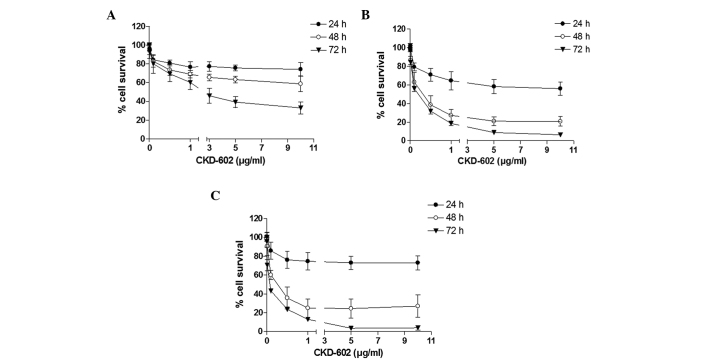
Cell viability following CKD-602 treatment. The cells were incubated with various concentrations of CKD-602 (0, 0.001, 0.01, 0.1, 1, 3, 5 and 10 μg/ml) for 24, 48 and 72 h. Cell viability was determined using an MTS assay. All cell lines exhibited a significant reduction in viability, dependent on CKD-602 treatment time and dose. (A) YD-8, (B) YD-9 and (C) YD-38 cell lines. The data are presented as the mean percentage of viable cells ± standard deviation (n=3).

Using the cell viability assay, CKD-602 was revealed to exert a significant cytotoxic effect on all cell lines in a time- and dose-dependent manner (Fig. 1). The cell viability IC_50_ values were 2.4 μg/ml for YD-8, 0.18 μg/ml for YD-9 and 0.05 μg/ml for YD-38 cells at 72 h following treatment (Table I).

### Effects of CKD-602 on the OSCC cell cycle

**Figure 2:**
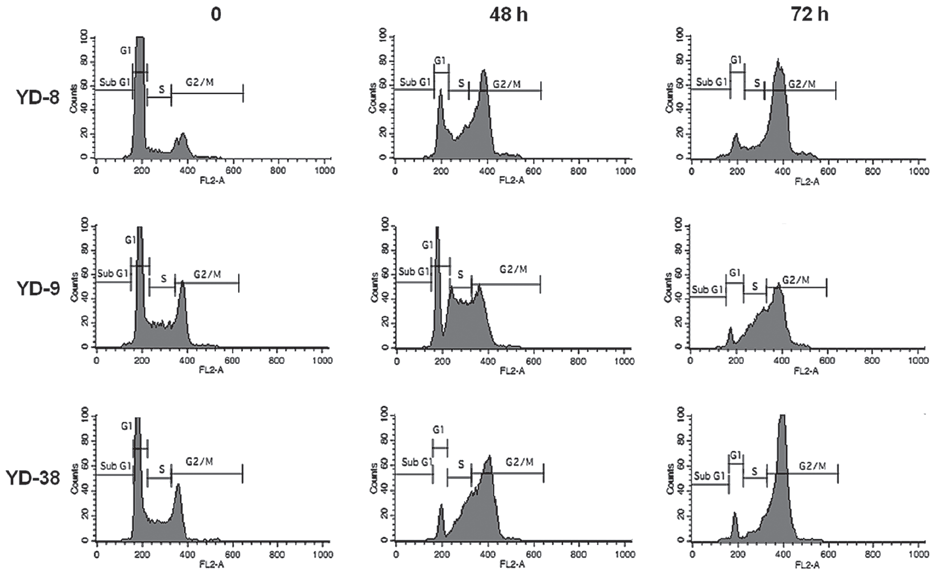
CKD-602 induces G2/M phase arrest in oral squamous cell cancer cells. The three YD cell lines were incubated with 0.02 μg/ml CKD-602 for 48 and 72 h, fixed and stained with propidium iodide, and analyzed for DNA content.

Cell cycle analysis was performed following treatment with 0.02 μg/ml CKD-602 for 48 h and 72 h in each cell line. CKD-602 induced G2/M phase cell accumulation in all cell lines (Fig. 2). The proportion of the cell population in G2/M phase at 72 h increased from 11.9±3.8 to 77.6±0.3% for YD-8 cells, from 25.2±3.6 to 54.0±5.4% for YD-9 cells and from 19.6±3.5 to 78.3±2.6% for YD-38 cells. The percentage of the cell population in the G1 phase at 72 h was reduced from 81.7±6.48 to 10.9±2.0% for YD-8 cells, from 49.4±4.5 to 7.3±1.56% for YD-9 cells and from 58.8±7.0 to 9.0±2.9% for YD-38 cells.

### Effects of CKD-602 on cell cycle regulatory protein expression levels

**Figure 3:**
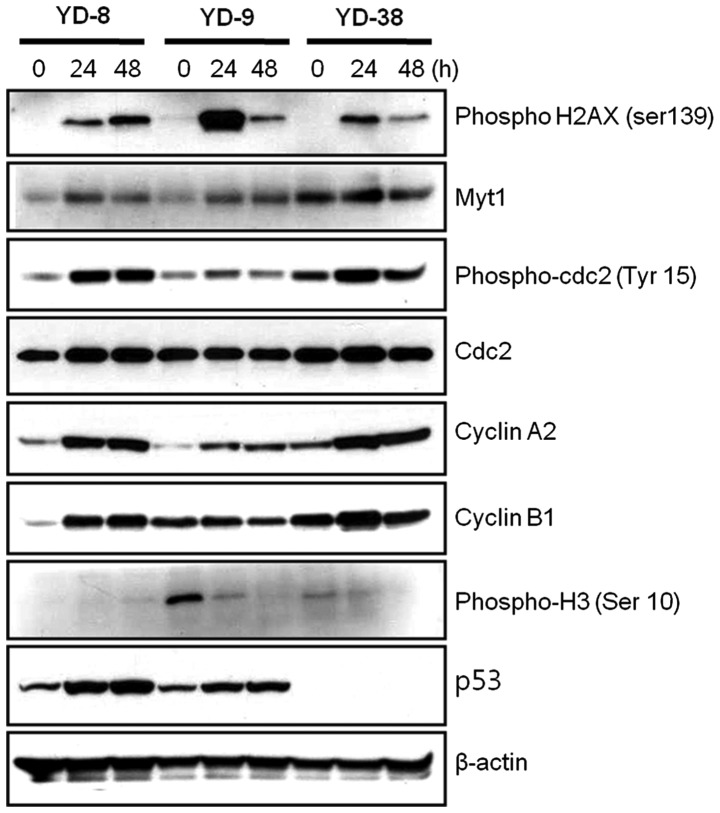
Effects of CKD-602 on cell cycle regulating molecules in YD cell lines. The cells were treated with 0.02 μg/ml CKD-602 and the protein expression levels at 24 and 48 h following treatment were examined by western blot analysis.

As CKD-602 induced G2/M phase arrest in the OSCC cell lines, whether alterations in the cell cycle regulatory proteins occurred following treatment with a non-cytotoxic dose of CKD-602 (0.02 μg/ml) was subsequently investigated. The phosphorylation of histone H2AX (γH2AX) Ser 139 is one of the early events initiated by double-stranded DNA breaks. Immunoblotting analysis revealed that the phosphorylation of histone H2AX was increased in the CKD-602 treatment cells. In the cell cycle, distinct cyclin/cyclin-dependent kinase complexes are activated to regulate cell cycle progression. Cyclin A- and cyclin B-associated cdc2 regulates the G2/M phases. During G2 phase, the cdc2/cyclin B complex is maintained as inactive by the phosphorylation of cdc2 Tyr 15 and Thr 14 by the Myt1 kinases (15–17). As shown in Fig. 3, the Myt1 protein expression levels in the three YD cell lines were observed to be increased at 24 h and reduced at 48 h CKD-602 treatment. The expression of cdc2, which is involved in cell cycle arrest in the G2 phase, was not significantly affected by CKD-602 as compared with the control treatment, however, the phosphorylated form, phospho-cdc2 (Tyr 15), was significantly increased at 24 h in all three YD cell lines, and reduced at 72 h in the YD-9 and YD-38 cells (P<0.05). Cyclin A2 protein expression levels were increased at 24 h and 48 h in the three YD cell lines subsequent to CKD-602 treatment as compared with the controls. Cyclin B1 protein expression levels were increased at 24 h following CKD-602 incubation in YD-8 and YD-38 cells, however, no change at 24 h was observed in the YD-9 cell line. These increases in cyclin B1 expression levels were reduced at 48 h following CKD-602 treatment in the YD-9 and YD-38 cell lines. p53 protein expression was detected by immunoblotting in the YD-8 and YD-9 cells, but not in the YD-38 cells (Fig. 3).

As the phosphorylation of histone H3 is a molecular checkpoint for entering mitosis (18), the phosphorylation of histone H3 Ser 10 following CKD-602 treatment was examined. As shown in Fig. 3, the phosphorylation of histone H3 Ser 10 was significantly reduced in a time-defendant manner in the YD-9 and YD-38 cell lines following CKD-602 treatment (P<0.05), however, this was not detected in the YD-8 cell line.

### Effects of CKD-602 on apoptosis in OSCC cell lines

**Figure 4:**
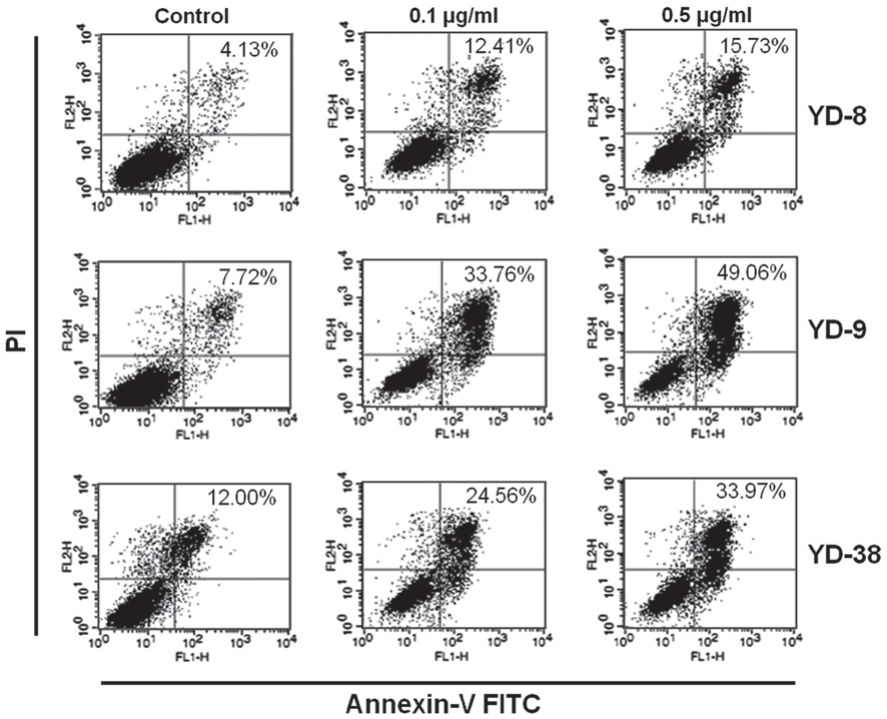
Induction of apoptosis in CKD-602-treated YD cell lines. The cells were harvested following 24 h incubation with 0.1 and 0.5 μg/ml CKD-602. Apoptosis was determined by staining the cells with Annexin V-FITC and PI labeling. The percentage figure in each window indicates the proportion of Annexin V-FITC positive cells. FITC, fluorescein isothiocyanate; PI, propidium iodide.

Annexin V/FITC staining, along with flow cytometry, enables the quantitative assessment of living (Annexin V-FITC-negative/PI-negative), early apoptotic (Annexin V-FITC-positive/PI-negative), late apoptotic/necrotic (Annexin V-FITC-positive/PI-positive) and dead (Annexin V-FITC-negative/PI-positive) cells. The effects of 24 h CKD-602 treatment on YD cell apoptosis are shown in Fig. 4 and Table II. The cells in the lower right quadrant indicate early apoptosis and the cells in the upper right quadrant signify late apoptosis. The proportion of Annexin-V/FITC-staining cells was markedly increased in a dose-dependent manner following treatment with CKD-602. However, the apoptotic proportion was lower in the YD-8 cells than in the YD-9 or YD-38 cell lines.

## Discussion

Topoisomerase inhibitors are a class of agents that target topoisomerase specifically by intercalating inside the topoisomeares cleavage complexes and mediate the changes in DNA structure during the normal cell cycle. Recently, topoisomerases have become widely investigated targets for cancer chemotherapy treatment. Topoisomerase inhibitors are hypothesized to suppress the regulatory step, which normally reseals the parent strand of DNA following passage of the daughter strand (19). The collision of the replication fork with the cleaved strand of DNA causes an irreversible double-stranded DNA break, which arrests the process of cell division and results in cell death (19).

CKD-602 is a potent Top1 inhibitor that successfully overcomes the poor water solubility and toxicity of the corresponding parent drug, camptothecin. Clinical trials for the treatment of various types of cancer with CKD-602 are ongoing and have shown promising results (13,14,20,21). CKD-602 may have potential in the treatment of patients with oral squamous cell carcinoma, as determined by the potent anticancer effects of the drug (14).

In the present study, CKD-602 induced cytotoxicity in OSCC cell lines, causing apoptosis in a dose- and time-dependent manner. The cytotoxic effect of CKD-602 was more prominent in the YD-9 and YD-38 cell lines, which did not possess p53 mutations, than in the YD-8 cell line, which did have a p53 mutation (Fig. 1 and Table I). Furthermore, the YD-9 and YD-38 cell lines exhibited more prominent apoptosis than the YD-8 cell line following CKD-602 treatment (Fig. 4 and Table II). Conflicting data have been reported concerning the influence of the p53 gene on the efficacy of topoisomerase inhibitors. Wang *et al* (22) demonstrated that a p53 disruption sensitizes glioblastoma cells to Top1 inhibitor-mediated apoptosis and wild-type p53 promotes a senescence-like phenotype, subsequent to SN-38 treatment. In another study, the cytotoxic effect of CKD-602 was more prominent in mutant p53 cell lines than in wild-type p53 cell lines (20). The involvement of p53 in the cytotoxic effects of anticancer agents remains under debate. Conflicting results have been generated using genetic models where p53 function has been modified by E6 protein expression (22,23) or homologous recombination (24), as cells with non-functional p53 may develop greater sensitivity, greater resistance or retain the same sensitivity, depending on the drug administered and the cellular context. Recent studies have refuted the role of p53 in determining differential susceptibilities to Top1 inhibitor (25,26). As determined by this evidence, the association between the p53 status and the effect of Top1 inhibitors, including CKD-602, requires further evaluation.

As over half of human tumors exhibit a p53 mutation or deficiency, the investigation of cell cycle checkpoints in tumor cells with various p53 statuses provides a potential basis for developing novel tumor therapeutics. In the present study, three OSCC cell lines were used with genetically different p53 statuses. The YD-8 cell line had a point mutation at codon 273 of exon 8 and the GGT sequence was altered to CAT, which resulted in a change from arginine to histidine. The levels of p53 protein were detected in YD-8 and YD-9 by immunoblotting (Fig. 3). As the half-life of wild type p53 protein is several minutes, p53 protein levels in normal cells are relatively low and are generally undetectable by immunoblotting. An abnormal p53 protein may be easily detected by immunoblotting due to the prolongation of half-life. However, the YD-9 cell line, which did not exhibit the p53 mutation, positively expressed the p53 protein (Fig. 3). Several possible underlying mechanisms, other than a point mutation, may result in the overexpression of the p53 protein (27,28). For example, genetic alternations in another region of the exon, such as the promoter or the intron of the p53 gene, may result in higher expression levels of the wild-type p53. The YD-38 cell line did not possess a p53 gene mutation and the p53 protein was not detected by immunoblotting (Fig. 3).

Treatment of the cells with CKD-602 for 48 h and 72 h resulted in cell cycle arrest at the G2/M phase (Fig. 2). This effect was associated with alterations in the expression of cyclins, including cyclin A and cyclin B1, molecules which provide molecular determinants for the cell cycle. This finding coincides with those from other studies with regard to the effect of CKD-602 on glioma cell lines. Kim *et al* (20) reported a reduction in the percentage of cells in the G1 phase and an increase in the percentage of cells in the G2/M phases in the U87 MG, U343 MG, U251 MG and LN229 human glioma cell lines following treatment with CKD-602.

In conclusion, in the present study, CKD-602 was demonstrated to exert an *in vitro* anticancer effect against OSCC cell lines by promoting cell cycle arrest in the G2/M phase and by inducing apoptosis. These findings suggest that CKD-602 is a promising candidate for use in oral cancer therapy and provides a rationale for the further evaluation of CKD-602 treatment in oral cancer by *in viv*o and clinical studies.

## Figures and Tables

**Table I:** Growth inhibition (IC_50_, μg/ml) of oral squamous cell cancer cell lines by CKD-602.

	IC_50_, μg/ml		
	
Treatment duration	YD-8	YD-9	YD-38
48 h	-	≥0.3	≥0.24
72 h	≥2.4	≥0.18	≥0.05

**Table II:** Results of FACS analysis evaluating apoptotic effects following treatment with CKD-602 for 48 h.

	Fraction of cells, %					
	
	YD-8		YD-9		YD-38	
			
CKD-602	Live	Apoptotic	Live	Apoptotic	Live	Apoptotic
Control	94.06±0.13	4.78±0.91	92.14±1.91	6.57±1.63	88.03±5.04	7.15±3.62
0.1 μg/ml	87.97±3.80	10.24±3.07	67.58±4.12	30.91±4.04	82.56±6.87	14.56±3.96
0.5 μg/ml	83.73±4.19	13.77±2.77	60.16±5.46	38.74±14.6	70.85±3.51	26.31±1.20
